# The Antidiabetic Effect of MSCs Is Not Impaired by Insulin Prophylaxis and Is Not Improved by a Second Dose of Cells

**DOI:** 10.1371/journal.pone.0016566

**Published:** 2011-01-27

**Authors:** Fernando Ezquer, Marcelo Ezquer, Valeska Simon, Paulette Conget

**Affiliations:** Instituto de Ciencias, Facultad de Medicina, Clinica Alemana Universidad del Desarrollo, Santiago, Chile; University of Bremen, Germany

## Abstract

Type 1 diabetes mellitus (T1D) is due to autoimmune destruction of pancreatic beta-cells. Previously, we have shown that intravenously administered bone marrow-derived multipotent mesenchymal stromal cells (MSCs) allows pancreatic islet recovery, improves insulin secretion and reverts hyperglycemia in low doses streptozotocin (STZ)-induced diabetic mice. Here we evaluate whether insulin prophylaxis and the administration of a second dose of cells affect the antidiabetic therapeutic effect of MSC transplantation. Insulitis and subsequent elimination of pancreatic beta-cells was promoted in C57BL/6 mice by the injection of 40 mg/kg/day STZ for five days. Twenty-four days later, diabetic mice were distributed into experimental groups according to if they received or not insulin and/or one or two doses of healthy donor-derived MSCs. Three and half months later: glycemia, pancreatic islets number, insulinemia, glycated hemoglobin level and glucose tolerance were determined in animals that did not received exogenous insulin for the last 1.5 months. Also, we characterized MSCs isolated from mice healthy or diabetic. The therapeutic effect of MSC transplantation was observed in diabetic mice that received or not insulin prophylaxis. Improvements were similar irrespective if they received one or two doses of cells. Compared to MSCs from healthy mice, MSCs from diabetic mice had the same proliferation and adipogenic potentials, but were less abundant, with altered immunophenotype and no osteogenic potential.

Our preclinical results should be taken into account when designing phase II clinical trials aimed to evaluate MSC transplantation in patients with T1D. Cells should be isolated form healthy donor, insulin prophylaxis could be maintained and a second dose, after an elapse of two months, appears unnecessary in the medium-term.

## Introduction

Diabetes mellitus is a complex metabolic disease with an estimated worldwide prevalence of 285 million cases in the adult population [1]. Among these, type 1 diabetes mellitus (T1D) represents 10% of the diabetic population and its increasing incidence in developed countries has generated a major global health issue [2].

T1D results from a cell-mediated autoimmune attack of pancreatic beta-cells [3]. At the time of clinical diagnosis, approximately 60% to 80% of the insulin-producing cells have been destroyed [4]. Standard strategies to care patients with T1D are based on careful monitoring of food intake and insulin prophylaxis. Nevertheless, a good metabolic control is difficult to reach and insulin therapy is frequently associated with severe episodes of hypoglycemia and hyperglycemia. Thus, even under treatment, patients with T1D develop severe long-term complications that clearly reduce their life expectancy [5], [6].

It has been shown that pancreatic islet transplantation restores normoglycemia in patients with T1D [7], [8]. However, limited availability of islet donors, high rates of graft failure and the need of life-long nonspecific immunosuppressive therapy of the receptor have been major obstacles to the widespread implementation of this treatment [9]. Therefore, a therapeutic alternative that can be broadly used is still needed. Among strategies under development, those based on stem cells are the most promising [10].

Multipotent mesenchymal stromal cells also referred as to mesenchymal stem cells (MSCs), is a population of self-renewable and undifferentiated cells present in several adult tissues [11], [12]. They represent an ideal therapeutic tool since they have a great potential to induce regenerative process [13]–[15]. MSCs can be easily isolated from bone marrow aspirates and rapidly expanded *ex vivo* [11], [16]. Both *in vitro* and *in vivo*, they differentiate into cells of mesodermal origin [11], but also into cells of non-mesodermal tissues [17]–[21]. In addition, MSCs can manage exacerbated immune response and has been reported as hypoimmunogenic cells, allowing allogeneic transplantation without the requirement of hystocompatibility, recipient conditioning and/or further immunosuppresion [15], [22]. Bone marrow-derived MSCs have been used in cell therapy strategies to treat patients with different diseases, with beneficial effect and without toxicity [23]–[26]. The antidiabetic therapeutic effect of MSCs has been tested on the preclinical models: NOD mice and streptozotocin (STZ)-induced diabetic murine [27]–[31]. The transplantation of syngeneic or allogeneic MSCs, alone or in association with hematopoietic stem cells, proved to be useful in preventing diabetes onset but also retarding its progression. Recently, we have shown that the intravenous administration of a single dose of 0.5×10^6^ MSCs to mice with T1D, results in the reversion of hyperglycemia and glycosuria [28]. This functional correction was associated with an increase in the number of pancreatic islets and with the recovery of the anatomical and numerical distribution of insulin- and glucagon-producing cells inside them. Thus, MSC transplantation allows pancreatic islet regeneration [27], [28]. Before translating these promising preclinical data, several practical issues should be addressed. Here we evaluate whether insulin prophylaxis and the administration of a second dose of cells affect the antidiabetic effect of MSC transplantation. Also, we characterized and compared MSCs isolated from mice healthy or diabetic. The rationale to test the former is that high-glucose culture conditions as well as *in vivo* hyperglycemia have been recognized as critical factors for the differentiation of adult stem cells into insulin-producing cells [32], [33]. Nonetheless, once diagnosed patients with T1D receive exogenous insulin to reduce they hyperglycemia. To our knowledge, nobody has evaluated the impact of MSC administration in a situation that resembles the stage at which patients with T1D will be treated, i.e. in normoglycemic diabetic receptors. By another hand, in the field of MSC transplantation is a matter of controversy whether multiple doses of cells have any beneficial impact. Finally, the best source of MSCs for transplantation is another unresolved issue. Autologous MSCs appear to be the ideal choice because they minimize infectious disease dissemination risk. However, chronic diseases could modify the abundance, the phenotype or the potentials of MSCs.

**Figure 1:**
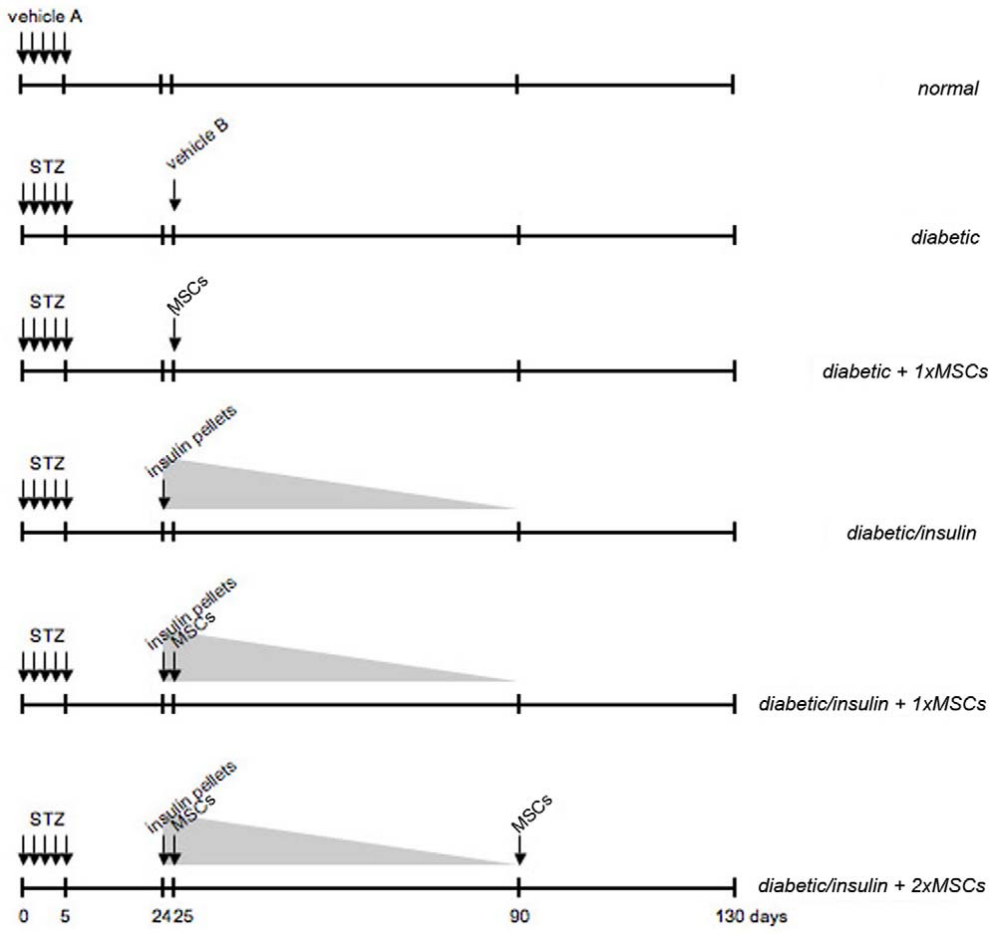
Experimental groups. Vehicle A: 0.1 M citrate buffer, pH 4.5. STZ: intraperitoneal 40 mg/kg/day, for five consecutive days. Vehicle B: 5% mice plasma. MSCs: intravenous 0.5×10^6^ healthy donor bone marrow-derived MSCs resuspended in 0.2 mL vehicle B. Insulin pellets: subcutaneous implantation of two timed-release porcine insulin pellets per animal. Triangle represents exogenous insulin levels.

To evaluate the desired variables, diabetes was induced in C57BL/6 mice by the administration of 40 mg/kg/day STZ for five consecutive days [34]–[36], [28]. Twenty-four days later, mice were randomly distributed into experimental groups according to if they received or not two timed-release porcine insulin pellets and/or one or two doses of 0.5×10^6^ bone marrow-derived MSCs that have been *ex vivo* expanded and characterized according to their expression of surface markers and differentiation potentials (Figure 1). Three and half months after the interventions: glycemia, pancreatic islet number, insulinemia, glycated hemoglobin level and glucose tolerance were determined in animals that did not received exogenous insulin for the last 1.5 months. Additionally, we characterized and compared the abundance, immunophenotype, proliferation and differentiation potentials of MSCs obtained from age-matched healthy and diabetic mice.

## Results

**Figure 2:**
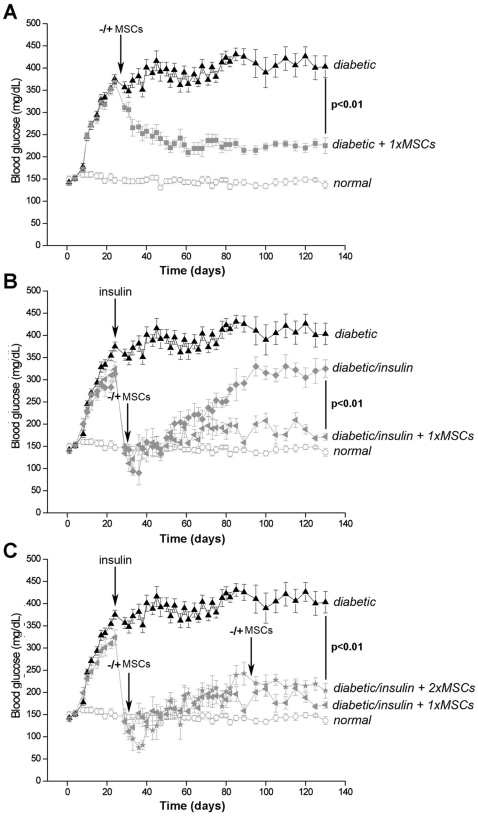
Hyperglycemia reversion in diabetic mice after MSC transplantation. Diabetic mice received or not MSCs (A). Diabetic mice received or not insulin prophylaxis and/or MSCs (B). Diabetic mice received or not insulin prophylaxis and one or two doses of MSCs (C). Blood glucose levels were determined every three days in alert nonfasted animals. Data correspond to mean ± s.e.m. for 9 animals per experimental group. Only statistical significant values among diabetic mice are shown.

**Figure 3:**
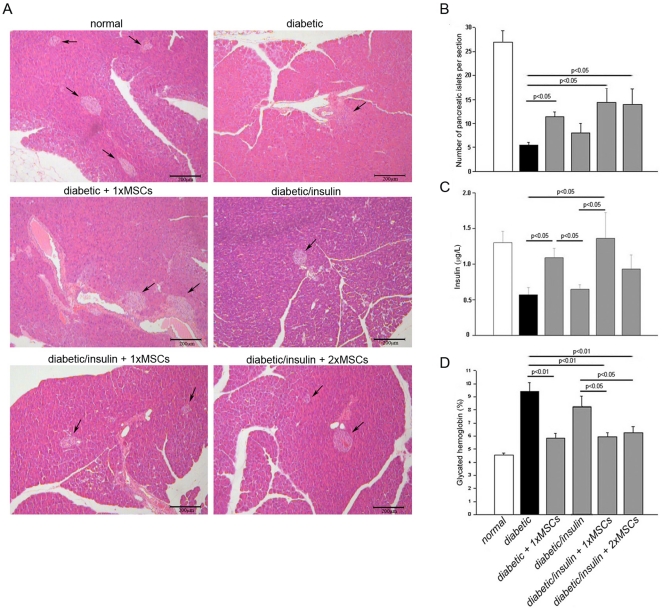
Pancreatic islets recovery, insulin secretion improvement and glycated hemoglobin normalization in diabetic mice after MSC transplantation. At day 130 after diabetes induction, pancreatic islets were observed (A) and quantified (B) in serial 4 µm hematoxilin/eosin–stained sections; mouse insulin levels were determined in venous blood samples obtained from alert fasted mice (C); glycated hemoglobin levels were determined in venous blood samples obtained from alert fasted mice (D). Qualitative data are representative from 4 animals per experimental group. Quantitative data correspond to mean ± s.e.m. from 9 animals per experimental group. Only statistical significant values among diabetic mice are shown.

Diabetes was induced in C57BL/6 mice by the administration of five low-doses of STZ. This protocol results in insulitis and in the destruction of pancreatic beta-cells [37], [34], [35]. As expected, animals gradually became hyperglycemic (*diabetic*, Figure 2) [28], [36], in association with a massive lost of pancreatic islets and hypoinsulinemia (*diabetic*, Figure 3) [28]. Without treatment diabetic mice survived for, at least, four months maintaining their body weight relatively constant and slightly increasing food and water intake.

Diabetic mice that received a single dose of MSCs when hyperglycemic progressively reduced their blood glucose levels (*diabetic + 1xMSCs*, Figure 2A). A month after MSC transplantation, those animals reached near euglycemic values (day 55, 228±30 mg/dL) that lasted, at least, for 3.5 months (day 130, 235±28 mg/dL). In contrast, untreated diabetic mice remained hyperglycemic until the end of the study period (day 130, 403±22 mg/dL) (*diabetic*, Figure 2A).

To evaluate whether MSC transplantation could induce the recovery of pancreatic islets in diabetic mice that were maintained normoglycemic by insulin prophylaxis, first we determined plasma exogenous insulin levels in diabetic mice that received subcutaneously two timed-release porcine insulin pellets. Exogenous insulin was found in animal plasma up to 45 days after pellet implantation (day 5, 29.7±0.8 mU/L; day 25, 25.4±0.9 mU/L; day 45, 18.3±0.8 mU/L); whereas at day 65, pellets were completely exhausted (not detectable). Accordingly, diabetic mice that received insulin prophylaxis on day 24 after STZ administration, six hours after pellet implantation became normoglycemic and remained in this condition until the exhaustion of the pellets (day 80, 180±16 mg/dL) (*diabetic/insulin*, Figure 2B). Thereafter, those animals began to increase their blood glucose levels reaching hyperglycemic values (day 130, 332±20 mg/dL). In contrast, diabetic mice that received insulin prophylaxis and a single dose of MSCs remained almost normoglycemic until the end of the study period (day 130, 175±12 mg/dL) (*diabetic/insulin + 1xMSCs*, Figure 2B).

We next evaluated whether the reduction of blood glucose level in diabetic mice transplanted with MSCs could be improved by the administration of a second dose of cells. For this, diabetic mice that received a first dose of MSCs under exogenously insulin-dependent normoglycemic condition were transplanted with a second dose of MSCs when insulin pellets were exhausted (Figure 1). No differences were observed in basal glycemic control between diabetic mice that received one or two doses of MSCs (day 130, 175±12 mg/dL vs. 197±23 mg/dL, respectively) (*diabetic/insulin + 1xMSCs* vs. *diabetic/insulin + 2xMSCs*, Figure 2C).

In experimental groups in which sustained hyperglycemia correction was attained (*diabetic + 1xMSC*s, *diabetic/insulin + 1xMSCs*, *diabetic/insulin + 2xMSCs*) a significant increase in pancreatic islet number (Figures 3A and B) and an improvement in endogenous insulin production (Figure 3C) were observed. Consequently, in the absence of exogenous insulin, diabetic mice that received MSCs restored their basal level of glycated hemoglobin (Figure 3D).

**Figure 4:**
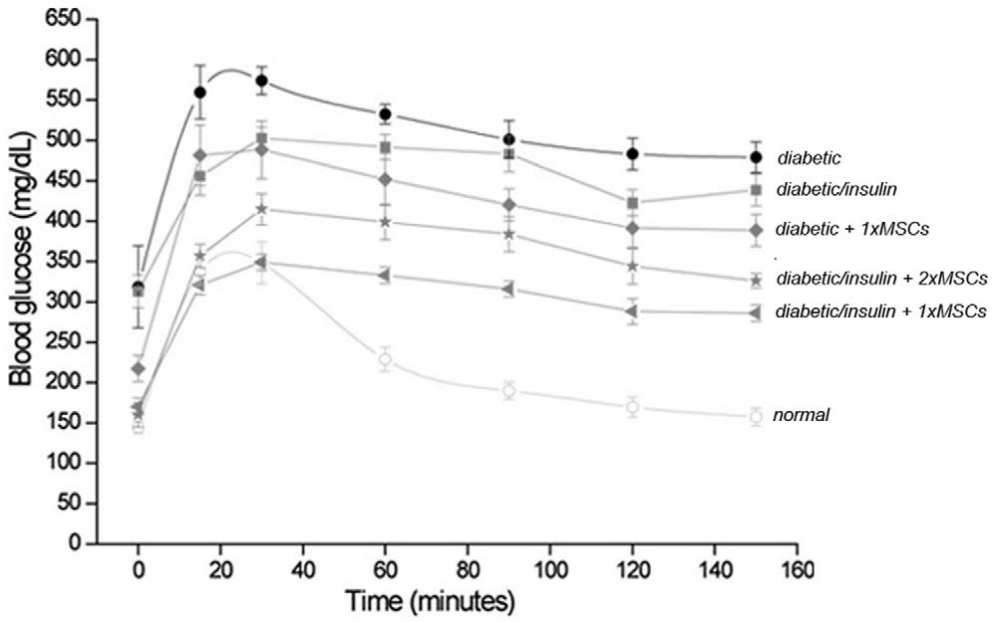
Glucose tolerance improvement in diabetic mice after MSC transplantation. At day 130 after diabetes induction, glucose tolerance was assessed in *normal*, *diabetic*, *diabetic + 1xMSCs*, *diabetic/insulin*, *diabetic/insulin + 1xMSCs* and *diabetic/insulin + 2xMSCs*. Data correspond to mean ± s.e.m. for 9 animals per experimental group. *p<0.05, diabetic vs. diabetic/insulin + 1xMSC*.

We also evaluated whether the recovery of pancreatic islets promoted by the transplantation of MSCs into diabetic mice contribute to the maintenance of normoglycemia under a massive glucose intake. For this, 130 days after diabetes induction mice were intraperitoneally injected with a high glucose solution and thereafter blood glucose levels were monitored. Glucose tolerance was slightly improved in diabetic mice transplanted with MSCs compared with untreated mice (*diabetic + 1xMSCs* vs. *diabetic*, Figure 4). The observed effect was statistical significant if the animals received insulin prophylaxis and MSCs (*diabetic/insulin + 1xMSCs*).

**Figure 5:**
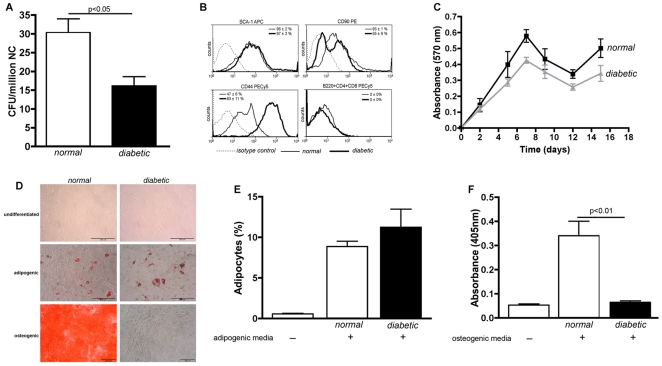
MSCs isolated from diabetic mice are exhausted. MSCs obtained from age-matched healthy mice (*normal*) or from mice 90 days after diabetes induction (*diabetic*) were characterized. Abundance in bone marrow was determined by CFU assay (A). Immunophenotyping was assessed by flow cytometry analysis after immunostaining with monoclonal antibodies against B220, CD4, CD8, SCA-1, CD90 and CD44 (B). Proliferation kinetic was evaluated according to crystal violet staining (Absorbance, 570 nm) during 15 days (C). Differentiation potentials were assessed after *in vitro* exposure to adipogenic or osteogenic medium and staining with Oil Red or Alizarin Red, respectively (D). Adipocytes generated were stained with Nile Red and quantified by flow cytometry (E). Matrix mineralization was quantified spectrophotometrically (Absorbance, 405 nm) after Alizarin Red staining (F). Qualitative data are representative from 4 animals per experimental group. Quantitative data correspond to mean ± s.e.m. from 4 animals per experimental group. Only statistical significant values are shown.

Finally, to approach to the best source of MSCs for transplantation we characterized cells isolated from mice 90 days after diabetes induction (*diabetic*) and compared them with MSCs obtained from age-matched healthy mice (*normal*). Viable MSCs were significantly more abundant in the bone marrow of normal than in diabetic mice (31±3 CFU/million nucleated cells vs. 16±2 CFU/million nucleated cells, respectively) (Figure 5A). MSCs isolated from healthy and diabetic mice did not express lymphocyte antigens (B220, CD4 and CD8) (Figure 5B). SCA-1 antigen expression did not varied between the cells of those animals. However, in diabetic mice half of the cells were negative for CD90 and all of them expressed high levels of CD44 (Figure 5B). MSCs obtained from diabetic mice showed the same proliferation potential as those obtained from normal mice (Figure 5C). Additionally, MSCs isolated from healthy mice could differentiate into both adipocytes and osteocytes, whereas MSCs obtained from diabetic mice could only differentiate into adipocytes (Figure 5D, E and F).

## Discussion

Despite the best efforts, there has been a steady increase in the number of patients with T1D worldwide [1]. Up to date, major goals are the regeneration of pancreatic beta-cells and the overcome of autoimmunity. In this context, MSC transplantation represents an interesting therapeutic option due to their potential to promote regeneration of several tissues and their immunomodulatory properties [13]–[15], [22].

Previously, we and other authors have proven that bone marrow-derived MSCs given intravenously contribute to the recovery of pancreatic islets in different diabetic animal models [27]–[31]. Nevertheless, before testing this cell therapy in patients with T1D, it is necessary to evaluate the impact of MSC transplantation in a situation that closely resembles the clinical scenario.

It has been proposed that *in vitro* and *in vivo* high glucose levels represent a critical factor for stem cell differentiation into insulin-producing cells [32], [33]. In addition, Kojima et al. showed that the hyperglycemia produced by a 25% glucose injection into non-diabetic mice is enough to lead to the appearance of insulin-positive cells in the liver, fat, spleen, bone marrow and thymus [33]. However, patients with T1D received insulin prophylaxis to maintain normoglycemia. Using a widely recognized mouse model of diabetes [34]–[37] here we demonstrate that, at both hyper- and normoglycemic conditions, MSC transplantation induces pancreatic islet recovery that results in an improvement endogenous insulin production. Therefore, insulin prophylaxis does not affect the antidiabetic effect of MSC transplantation; indicating that the success of this cell therapy strategy is independent of receptor glucose level. Observed hyperglycemia correction correlates with the normalization of glycated hemoglobin levels, demonstrating that MSC transplantation allows the maintenance of blood glucose at steady levels and prevents episodes of hypoglycemia or severe hyperglycemia. Furthermore, an improvement in glucose tolerance was observed after MSC transplantation. Hence, MSC-induced pancreatic islet recovery, although incomplete, seems to be sufficient to regress diabetic mice into a pre-diabetic stage because they manage blood glucose level properly under a standard glucose diet but only partially under glucose overload. The phenotypic corrections here describe were not improved after the administration, two months later, of a second dose of MSCs.

In animals that received MSCs, we found no solid tumor at the necropsy. This result was not unexpected, since we transplanted cells at passage 3 and tumor formation seems to be associated to the administration of MSCs heavily expanded *ex vivo* [38].

Up to date, the molecular mechanisms behind the antidiabetic effect of MSC transplantation have not been unequivocally proven. The contribution of trophic factors produced by MSCs [39], [40] may be relevant because it has been shown that HGF and IGF-1 prevent the apoptosis and stimulate the proliferation of pancreatic beta-cells [41]–[43]. On the other hand, MSCs can modulate the immune responses by the release of regulatory cytokines that inhibit dendritic cells [44], suppress autoreactive T cells [15] or promote a shift toward a protective Th2 response [31]. Hence, in diabetic mice the administration of a single dose of MSCs seems to promote pancreatic islets regeneration and restrict autoimmunity.

In order to provide evidence supporting the best source of MSCs for transplantation, we compared the characteristic of cells obtained from the bone marrow of age-matched healthy animals with that of diabetic mice. Autologous MSCs are the most attractive candidate because they are devoid of infectious disease dissemination risk. It has been proven that MSCs from patient with T1D can be isolated, expanded and differentiate into insulin-producing cells [45], [46]. Here, we show that viable MSCs obtained from diabetic mice have the same proliferation and adipogenic potentials than that of age-matched healthy mice. However, the abundance, surface marker expression and osteogenic potential are different between MSCs isolated from diabetic and normal mice. Accordingly, Stolzing et al. recently demonstrated that the number of colonies and the osteoblastic differentiation potential of MSCs isolated from STZ-induced diabetic rats are significantly reduced compared with that obtained from age-matched healthy animals [47]. Also, it has been reported that MSCs isolated from diabetic mice exhibit high gene expression level of senescent (*p16^INK4^, p66^shc^, p53*) and apoptotic (*Bax, Bak*) markers and down-regulated the expression of survival genes (IGF-1, FGF-2, Akt) [48]. The differences observed by us between MSCs isolated from healthy control and diabetic mice seems not to be related to STZ direct toxicity because: i) the *in vivo* clearance of STZ is very fast [49], ii) MSCs isolated seven days after the administration of a high dose of STZ to rats show the same characteristics of MSCs isolated from healthy rats [47], iii) we isolated MSCs 90 days after STZ administration. On the other hand, the observed differences seems to be related to the diabetic process because: i) Stolzing et al. reported that at least four weeks of severe hyperglycemia are necessary to observe some alterations in MSCs [47], ii) most of the alterations observed in MSCs isolated from diabetic mice could also be evidenced when MSCs isolated from healthy animals were incubated in high glucose medium that emulates *in vitro* a hyperglycemic condition [48], [50], iii) we isolated MSCs from mice that maintained hyperglycemia higher than 400 mg/dL for most than two months. Hence, the exhaustion of MSCs observed in our diabetic mice seems to be related not to STZ treatment but to the diabetic condition. Considering the altered phenotype of MSCs isolated from diabetic mice, and the fact that MSCs isolated from NOD mice were unable to delay diabetic onset [31], we decided not to evaluate their antidiabetic effect *in vivo*. Thus, allogeneic bone marrow seems to be the ideal source of MSCs for transplantation in patients with T1D, as is the case in the treatment of patients with other diseases, where promising results and no toxicity has been described [51], [52].

Our preclinical results should be taken into account when designing phase II clinical trials aimed to evaluate MSC transplantation in patients with T1D. Cells should be isolated form healthy donor, insulin prophylaxis could be maintained and a second dose of MSCs, after an elapse of two months, appears unnecessary in the medium-term.

## Materials and Methods

### Animals

C57BL/6 mice (Jackson Laboratory, Bar Harbor, ME) were housed at constant temperature and humidity, with a 12∶12 hours light-dark cycle and unrestricted access to a standard diet and water. When required, animals were lightly anesthetized with sevofluorane (Abbott, Japan). Animal protocols were approved by the Ethic Committee of Facultad de Medicina, Clinica Alemana Universidad del Desarrollo. Approval ID # 010-2008.

### Diabetes induction

Eight week-old male C57BL/6 mice were lightly anesthetized. STZ (Calbiochem, La Jolla, CA) was dissolved in vehicle A (0.1 M citrate buffer, pH 4.5), and immediately injected intraperitoneally at a dose of 40 mg/kg/day, for five consecutive days [28].

### Blood glucose determination and diabetes diagnostic criteria

From non-fasted alert animals, blood samples were collected from the tail vein and glucose levels were determined with the glucometer system Accu-Chek Go from Roche Diagnostic (Mannheim, Germany). Mice were considered diabetic if blood glucose levels were above 250 mg/dL, on three consecutive determinations.

### MSC isolation and *ex vivo* expansion

Six to eight week-old C57BL/6 mice were sacrificed. Bone marrow cells were obtained by flushing femurs and tibias with sterile PBS. After centrifugation, cells were resuspended in alpha-MEM (Gibco, Auckland, NZ) supplemented with 10% selected fetal bovine serum (Gibco, Auckland, NZ) and 80 mg/mL gentamicin (Sanderson Laboratory, Chile) (alpha-10) and plated at a density of 1×10^6^ nucleated cells/cm^2^. Non-adherent cells were removed after 72 hours by media change. When foci reach confluence, adherent cells were detached with 0.25% trypsin, 2.65 mM EDTA (Gibco, Auckland, NZ), centrifuged and subcultured at 7,000 cells/cm^2^. After two subcultures, adherent cells were characterized and transplanted.

### MSC characterization

*CFU assay*: Bone marrow cells were resuspended in alpha-10 and plated at a density of 17,000 nucleated cells/cm^2^ in triplicate. Non-adherent cells were removed by media change twice a week. At day 21, cells were fixed with 4% paraformaldehyde for 10 minutes and stained with 0.5% crystal violet (Sigma-Aldrich, St. Louis, MO) in 10% methanol for 20 minutes. After four washes, colonies formed by more than 30 fibroblast-like cells were counted under light microscope at low magnification. Results were expressed as CFU/million of nucleated cells plated.

#### Immunophenotyping

Although there are currently no consensus markers for murine MSCs as there exist for human MSCs [12], immunophenotyping was performed by flow cytometry analysis after immunostaining with monoclonal antibodies against lymphocyte markers B220, CD4, CD8 (PE-Cy5-conjugated, BD Pharmingen, San Jose, CA) and putative murine MSC markers SCA-1 (APC-conjugated), CD90 (PE-conjugated) and CD44 (PE-Cy5-conjugated) (all from eBioscience, San Diego, CA).

#### Proliferation assay

First passaged MSCs were subcultured at 4,000 cells/cm^2^. Media was changed every three days. Cell number was determined at days 0, 2, 5, 7, 9, 12 and 15 after staining with 0.5% crystal violet in 10% methanol for 20 minutes. After four washes, cell-incorporated crystal violet was solubilized by incubation with phosphate buffer in methanol (50∶50) and quantified spectrophotometrically (absorbance, 570 nm).

#### Differentiation assays

MSCs were incubated with standard adipogenic or osteogenic differentiation media for 14 and 21 days, respectively [53]. To quantify the adipogenic potential, cultures were stained with 1 mg/mL Nile Red (Sigma-Aldrich, St. Louis, MO) and analyzed by flow cytometry [54]. To quantify the osteogenic potential, cultures were fixed with 10% formaldehyde and stained with 40 mM Alizarin Red (Sigma-Aldrich, St. Louis, MO). After four washes, Alizarin Red stain was solubilized with 10% acetic acid neutralized with 10% ammonium hydroxide and quantified spectrophotometrically (absorbance, 405 nm) [55].

### MSC transplantation

0.5×10^6^ MSCs were resuspended in 0.2 mL of vehicle B (5% mice plasma) and administered via the tail vein to lightly anesthetized mice.

### Insulin prophylaxis

Twenty-four days post-diabetes induction, two timed-release porcine insulin pellets (Linbit, Linshine, Canada) were implanted subcutaneously, following manufacturer instructions, to lightly anesthetized mice.

### Pancreas histology

Mice were sacrificed by cervical dislocation. Pancreas were rapidly removed, fixed in 4% paraformaldehyde and embedded in paraffin. Four µm pancreatic sections were stained with hematoxilin-eosin (Sigma-Aldrich, St. Louis, MO). Slices were analyzed under light microscopy, focusing on pancreatic islets. Images were captured with a digital camera.

### Plasma insulin determination

Plasma insulin was assayed on blood samples collected from the tail vein of fasted alert animals using mouse or porcine insulin ultrasensitive ELISA kits (Mercodia, Uppsala, Sweden). Kit reagents guarantee no cross-reactivity between those insulins.

### Glycated hemoglobin determination

HbA_1c_ was assayed on blood samples collected from the tail vein of fasted alert animals using DCA2000 analyzer (Bayer Corporation, Elkhart, IN) [56].

### Intraperitoneal glucose tolerance test

After six hours of fasting, mice were lightly anesthetized and injected intraperitoneally with 2 mg glucose/g body weight. Blood glucose levels were determined 15 minutes before and 15, 30, 60, 90, 120 and 150 minutes after glucose administration. Area under the curve was determined by the trapezoidal rule.

### Statistical analysis

Data shown correspond to all animal studied and were represented as mean±s.e.m. Multiple group comparisons were performed by ANOVA followed by Bonferroni post-hoc test. p<0.05 were considered statistically significant.
